# Stem cells harvested from different sources ameliorate liver fibrosis: comparative study

**DOI:** 10.1186/s13287-025-04707-6

**Published:** 2025-10-30

**Authors:** Islam S. Ali, Nadeen D. Abdel-Wahab, Ahmed Nabil

**Affiliations:** 1 Basic Science Department, Delta University for Science and Technology, Gamasa, 35712 Dakahlia Egypt; 2 Department of Biochemistry and Molecular Biology, Faculty of Veterinary Medicine, Mansoura University, Mansoura, 35516 Egypt; 3 Department of Biotechnology and Life Sciences, Faculty of Postgraduate Studies for Advanced Sciences (PSAS), Beni-Suef University, Beni-Suef, 62521 Egypt

**Keywords:** Liver fibrosis, Thioacetamide, BM-MSCs, AD-MSCs, L-MSCs

## Abstract

Hepatic fibrosis is a serious illness that can lead to death. Until recently, there has been no effective medication to protect the liver and heal fibrosis. When thioacetamide (TAA)-induced hepatotoxicity occurred, we aimed to determine the hepatoprotective effectiveness of adipose, bone marrow, and liver-derived mesenchymal stem cells. Fifty male albino Wistar rats were used throughout the study and divided into 5 groups. Each group had 10 rats divided into 2 cages, 5 rats per cage. Forty male albino Wistar rats were injected intraperitoneally with 100 mg/kg of thioacetamide 2 times a week for 9 weeks. After 5 weeks of induction of liver fibrosis, forty rats were divided into four groups. One group was considered the TAA group and didn’t receive any treatment, and the other three groups were inoculated with a single dose of 3 × 10^6^ BM-MSCs, AD-MSCs, and L-MSCs, respectively. We assessed the liver function tests as ALT, AST, and total bilirubin, which showed a significant decrease in groups inoculated with stem cells. But although the most significant decrease appeared in the L-MSCs inoculated group. Additionally, there was a notable rise in albumin levels in the L-MSC-inoculated group. In groups injected with stem cells, namely L-MSCs, the evaluation of antioxidant and oxidative stress levels revealed a substantial increase in GSH concentration, SOD activity, and CAT activity. Additionally, there is a noticeable drop in MDA and NO levels. By the ELISA technique, we assessed the hydroxyproline and collagen type-1, and the results showed a significant decrease in the groups inoculated with stem cells, especially L-MSCs. As well, this group showed a significant downregulation of ASMA gene expression. The percentage of fibrosis assessed in the histopathologic samples showed the most significant decrease in the L-MSCs inoculated group. In conclusion, our study showed that the different types of mesenchymal stem cells had an ameliorative effect on hepatic fibrosis, but the best results appeared with L-MSCs rather than BM-MSCs and AD-MSCs. The treated group with L-MSCs showed great enhancement in the antioxidant status, decreased hydroxyproline and collagen type-1 content, and a lower percentage of fibrosis.

## Background

An excessive amount of extracellular matrix (ECM) constituents causes scar tissue, which is a hallmark of liver fibrosis, a degenerative wound healing response [1]. Liver fibrosis has several causes, including ischemic damage, congenital abnormalities, viral or autoimmune hepatitis, alcohol misuse, non-alcoholic steatohepatitis (NASH), and non-alcoholic fatty liver disease (NAFLD) [2, 3]. Hepatocellular carcinoma, liver cirrhosis, and irreversible end-stages of liver failure can develop from untreated liver fibrosis [4]. In the majority of cases, liver transplantation is typically advised since treating the condition would not be entirely successful with either eliminating the etiology or using antifibrotic medications [5].

The development of liver fibrosis is influenced by several signaling pathways and results from intricate interactions between various hepatic cells [5]. Hepatotoxic substances impact the liver's hepatocytes and cause them to undergo apoptosis. Further activating hepatic stellate cells (HSCs) and promoting the invasion of inflammatory cells, including apoptotic bodies and reactive oxygen species (ROS) [6]. A variety of inflammatory chemicals are released by inflammatory cells, which cause the liver to remain inflamed and encourage HSCs to make collagen [7]. Hepatic macrophages, including monocyte-derived or Kupffer cell (KC)-derived macrophages, fall into two categories: immunoregulatory macrophages (M2) and pro-inflammatory macrophages (M1) [8]. They can keep HSCs in their activated state and accelerate the progression of hepatic fibrosis [9].

Elevated ECM level is a hallmark of liver fibrosis, and activated HSCs create the majority of its constituents [10]. In the healthy liver, HSCs also make collagen and other extracellular matrix proteins, but in liver fibrosis, they undergo a transformation that results in a phenotype similar to that of myofibroblasts [11]. Activated HSCs display smooth muscle actin (SMA), develop contractile, proinflammatory, and fibrogenic characteristics, and lose their capacity to retain vitamin A droplets [12, 13]. When liver damage occurs, the amount of extracellular matrix (ECM) can increase by up to six times compared to normal [14]. There is an abundance of fibronectin, vimentin, elastin, laminin, and collagens I, III, and IV, and tissue inhibitors of MMPs (TIMPs) are expressed more often, whereas metalloproteinases (MMPs) have decreased activity [15, 16].

Several signaling pathways are crucial to the development of liver fibrosis, including the activation of HSCs. Platelet-derived growth factor (PDGF) is produced by KCs and HSCs that have been activated [17]. In HSCs, this component may also activate other signaling pathways, including the Ras/Raf, PI3K/Akt, and JAK/STAT pathways. Collagen I, MMPs, and TIMPs are examples of fibrotic indicators that will be produced under the direction of these pathways [18].

The organosulfur material thioacetamide (TAA), a well-known liver-toxic agent that damages the liver, produces free radicals, and induces inflammation [19]. It is used in labs and a variety of industries, including beverage, automobile fuel, paper, textile, leather, pesticides, rubber auxiliary products, and as a food additive and fungicide [20]. Toxic fumes, contaminated water, skin contact, or exposure to sewage are some of the ways that TAA can enter a human body [21]. Tissue damage occurs in several body organs as a result of this exposure. After extended exposure to TAA, hepatic tissues initially develop fibrosis and then cirrhosis [22]. TAA is an excellent model for assessing prospective anti-fibrotic treatments since it has been used by a number of previous researchers to induce hepatic tissue damage in laboratory animals, which mimics the hepatic fibrosis in people [23].

To encourage tissue repair, stem cell treatment is a substitute for liver transplantation. The potential for regeneration has been studied in a variety of cell sources, including hepatocytes [24] and several stem cell types [25, 26]. Stem cells fall into two main categories: adult stem cells (ASCs) and embryonic stem cells (ESCs). Both induced pluripotent stem cells (iPSCs) [27] and ESCs [28–30] are ways to obtain stem cells with pluripotency that can differentiate into cells that resemble hepatic cells. As multipotent stem cells, ASCs are less able to differentiate into diverse types of cells than ESCs. ASCs examined for liver regeneration include mesenchymal stem cells (MSCs) [31, 32] and liver stem cells (LSCs) [33, 34] from various tissue sources, including peripheral blood, cartilage, bone marrow (BM-MSCs) [35], adipose tissue (ADSCs) [36–38], and umbilical cord stem cells (UC-SCs). MSCs are the ideal choice for liver regeneration since they are freely accessible, self-renewing, and have minimal immunogenicity and may be employed without raising ethical concerns [39]. They may respond to signals of cellular damage by migrating to injury sites, and it has been demonstrated that they encourage the migration of other cells to liver locations, develop into cells like liver cells, and take part in hepatocyte regeneration [40–42].

Mesenchymal stem cell (MSC) treatments have become a cutting-edge option for the management of advanced liver fibrosis. Animal model studies have demonstrated that fulminant hepatic failure may be reversed [43–45], and liver fibrosis can be improved by infusing bone marrow-derived MSC (BM-MSC) [46, 47]. Autologous BM-MSC infusions have been shown in several trials to significantly improve hepatic performance in individuals with cirrhosis of the liver in a clinical setting [48, 49]. Specifically, it has been shown that BM-MSC infusions are both safe and practical for treating liver failure [50, 51]. Additionally, a study discovered that autologous BM-MSC treatment was safe for enhancing liver function and histological fibrosis in alcoholic cirrhosis patients [52].

The objectives of this study were to use stem cells as a novel way to promote tissue repair, explore the possibility that different mesenchymal stem cells could slow down the progression of liver tissue fibrosis caused by TAA in Albino Wistar rats, a model that mimics chronic liver damage in humans, and elucidate the fundamental ideas underlying their effects on liver function tests like ALT activity, AST activity, albumin, and total bilirubin concentrations. NO, MDA and GSH concentrations, as well as CAT and SOD activity, indicate the antioxidant state. ASMA expression, the amount of hydroxyproline, and collagen type 1.

We assumed that MSCs derived from bone marrow, liver, and adipose tissue would attenuate thioacetamide-induced liver fibrosis in rats, with liver-derived MSCs predicted to produce more therapeutic outcomes in comparison with the other MSC sources.

## Materials and methods

### Materials

Thioacetamide and corn oil were purchased from Sigma Aldrich. The minimum essential medium (MEM) supplemented with FBS and 1% penicillin/streptomycin (Pen/Strip), as well as phosphate buffer saline (PBS), was purchased from Thermo Fisher Scientific. Kits for liver function tests (ALT, AST, ALB, and total bilirubin) had been bought from BIOMED MEDICAL ANALYSIS. Kits for oxidative stress markers (SOD, MDA, CAT, NO, GSH), as well as ELISA kits for collagen type 1 and hydroxyproline content, were purchased from Sigma Aldrich. The PCR kits for ASMA level had been bought from OpGen.

### Methods

The work has been reported in line with the ARRIVE guidelines 2.0.

#### Isolation of adipose, bone marrow, and liver-derived mesenchymal stem cells

##### Isolation of BM-MSCs (Bone marrow mesenchymal stem cells)

The albino Wistar rats used for stem cell isolation were 8–10 months of age and weighed 180–200 g. The femur and tibia of the rat's hind leg were collected for the BM-MSCs isolation. Both the femur and the tibia were cleaned twice with PBS containing streptomycin and penicillin after the removal of the peripheral muscle tissue. A bone clamp was used for the breakdown of the epiphyseal at both ends, and α-MEM + FBS was used to flush the bone marrow to the outside. A 200-mesh filter was used to clarify the bone marrow suspension, and centrifuged. The cells were resuspended in a growth medium and covered with T25 flasks, then incubated at 37 °C and 5% CO_2_. The initial medium refreshment was completed after 48 h, and the medium was thereafter replaced every 3 days until the cells achieved an 80%–90% confluence [53].

##### Isolation of AD-MSCs (Adipose mesenchymal stem cells)

The albino Wistar rats used for stem cell isolation were 8–10 months of age and weighed 180–200 g. The rat's bilateral inguinal adipose tissue was used to collect AD-MSCs, which were then briefly submerged in alcohol before being double-washed with phosphate-buffered saline (PBS) containing streptomycin and penicillin. Then, the adipose tissue was cut into small fragments and preserved in collagenase II with shaking at 120 rpm in an incubator at 37 °C. Adipose tissue fragments turned into a fine mist after 60 min. To stop the digestion, a growth medium containing fetal bovine serum (FBS) was then added. The adipose tissue suspension had been filtered through sieves before being centrifuged for 5 min at 1500 rpm to get cell pellets made up of stromal vascular cells. In PBS, the pellet was reconstituted, and following coating of cells with FBS and the minimum required media (MEM) in T25 flasks, the cells were cultured at 37 °C with 5% CO_2_. The media was initially replaced after 48 h and then every 3 days until the cells achieved an 80–90% confluence [54].

##### Isolation of L-MSCs (Liver-derived mesenchymal stem cells)

The albino Wistar rats used for stem cell isolation were 8–10 months of age and weighed 180–200 g. Two grams of the liver from a Wistar rat were used to isolate L-MSCs, which were thoroughly cleaned by using phosphate buffer saline. The chunk of liver was pulverized and treated for 60 min in collagenase before being incubated for 45 min in trypsin. To get rid of any remaining blood, the cells were then centrifuged twice for 10 min. After being kept in T25 flasks and dissolved in FBS and DMEM with antibiotics, the cell pellet was incubated at 37 °C with 5% CO_2_. Until the cells achieved 80% to 90% confluence, the medium was replaced every 3 days following the first medium refreshment, which was completed within 48 h [55].

### Experimental design and ethics statement

The entire experimental protocol was approved by Mansoura University's Animal Care and Use Committee (MU-ACUC), under code: MU-ACUC (VM.R.24.11.192).

Fifty male albino Wistar rats were used. They weighed 200 g and were 8–10 weeks of age and were purchased from the Egyptian Organization for Biological Products and Vaccines (VACSERA, Giza, Egypt). All animals included in the experiment were healthy, and those that began the experiment completed the full duration of the study. They were divided into 5 groups; each group contained 10 rats. Rats were accommodated in a controlled environment with a 12-h dark/light cycle, a relative humidity of 50%, and a temperature of 21 ± 2 °C. To allow the rats to acclimate to their new surroundings, they were housed for a week prior to the start of the study. Rats were kept in cages with a consistent environment and diet for the duration of the trial (5 rats per cage). Cages were randomly arranged on the cage racks and alternated routinely throughout the study to avoid bias due to cage placement. Treatments and sample collections were accomplished in a fixed order and by the same operator to reduce personnel-related variation.

No formal a priori sample size calculation was conducted; however, the group size (n = 10) was determined based on a previous study using similar experimental designs. This size was sufficient to allow for statistical analysis and limit animal use following the ethical standards [56].

All animals and data points from each experimental group were included in the final analysis. No animals or data points were excluded from any group.

### Inoculation of AD-MSCs, BM-MSCs, and L-MSCs into rats to improve the TAA-induced liver fibrosis

The rats were anaesthetized by inhaling isoflurane to be painless for injection of thioacetamide and inoculation of the various stem cells. Each of the five main groups of 10 rats that made up the experimental animals was assigned at random in a way to guarantee unbiased distribution among the groups and matched for body weight and age as the following manner: Group I (control group): rats received twice-weekly intraperitoneal injections of 1 ml/kg maize oil for nine weeks. Group II (TAA group): For nine weeks, rats received two intraperitoneal injections of 100 mg/kg of thioacetamide [57]. Group III (Thioacetamide + BM-MSCs): Rats were intraperitoneally injected with 100 mg/kg of thioacetamide 2 times a week for 9 weeks, and a single dose of 3 × 10^6^ BM-MSCs was inoculated in the rats' tail vein at the fifth week of fibrosis induction [58]. Group IV (Thioacetamide + AD-MSCs): Rats were injected with 100 mg/kg of thioacetamide twice a week for 9 weeks, and a single dose of 3 × 10^6^ AD-MSCs was inoculated in the rats' tail vein at the fifth week of fibrosis induction [59]. Group V (Thioacetamide + L-MSCs): Rats were intraperitoneally injected with 100 mg/kg of thioacetamide 2 times a week for 9 weeks, and a single dose of 3 × 10^6^ L-MSCs was inoculated in the rats' tail vein at the fifth week of fibrosis induction [60]. All rats were given intraperitoneal thiopental (150 mg/kg) to render them unconscious at the end of the experiment. Moreover, the same method was employed to render the rats used to isolate BM-MSCs, AD-MSCs, and L-MSCs unconscious [61]. The blood samples were collected from the retro-orbital sinus to separate the serum, and rats were cervically dislocated and dissected to collect tissue samples.

### Assessment of liver function tests

The blood samples were taken from the retro-orbital sinus, and they were tilted for 30 min so that centrifugation at 3000 rpm for 10 min would separate non-hemolyzed serum.

### Evaluation of ALT enzyme activity in serum

The serum samples and reagents, which are R1, contained Good’s Buffer, L-Alanine, and LDH (lactate dehydrogenase enzyme), and R2 contained NAD and oxoglutarate, were at room temperature before use. To perform the test, we mixed 4 parts of R1 to 1 part of R2, then mixed them with the serum specimen, and the initial absorbance at 365 nm wavelength was taken after 60 s., and then the timer started simultaneously, and we took another reading after one, two, and three minutes. The average change in absorbance per minute was calculated (∆A/min) [62].

### Evaluation of AST enzyme activity in serum

The serum samples and reagents, which are R1, contained phosphate buffer and L-aspartate, and R2 contained LDH (lactate dehydrogenase), MDH, NADH, and oxoglutarate, were at room temperature before use. To perform the test, we mixed 4 parts of R1 to 1 part of R2 and then mixed them with the serum specimen, and the initial absorbance at 365 nm wavelength was taken after 60 s., and then the timer started simultaneously, and another reading was taken after 1, 2, and 3 min. They calculated the average change in absorbance each minute (∆A/min) [62].

### Determination of total bilirubin concentration in serum

The serum sample was mixed with R1, which contained sulfanilic acid and HCl; R2, which contained sodium nitrite; and R3, which contained caffeine and sodium benzoate, and was kept at 20–25 °C for 10 min. R4, which included sodium hydroxide and tartrate, was then added, combined, and incubated at 20–25 °C for five minutes. At 578 nm (560–600 nm), the absorption value of the specimen (A sample) in comparison to the standard was measured [63].

### Determination of albumin concentration in serum

The serum sample was mixed with R2, which contained buffer pH 3.8 and bromocresol green, and incubated for 5 min at room temperature (+ 15–25 °C). Then the absorbance of standard and sample tubes was taken at 623 nm [64].

### Evaluation of oxidative stress markers

The liver tissues were collected, weighed, and homogenized with cold PBS in a 1:9 ratio; tissue was ground using sterile sand using a mortar and pistol; samples were centrifuged for 10 min at 4000 rpm, the supernatant was collected for measurement of the oxidative stress parameters, and the sediment was removed before being stored at -80 for additional analysis.

### Determination of nitric oxide (NO) concentration

NaOH (0.3 N) was mixed with the liver homogenate. After 5 min of incubation at room temperature to deproteinize, zinc sulfate was added. After centrifuging the prior combination, the supernatant was collected and combined with HCl and VCl_3_ (8 mg/ml) in Griess reagent. Spectrophotometric measurements of the samples were made at 540 nm. Using the NaNO_3_ standard curve, the nitric oxide levels in the samples were determined [65].

### Determination of malondialdehyde (MDA) concentration

To the liver homogenate, 1-methyl-2-phenyl-indole was added to acetonitrile that had been diluted with FeCl_3_ + methanol. Samples were well mixed after 37% HCl was added. A tight stopper was used to seal the samples. Following a 10-min centrifugation and cooling in ice, samples were measured for optical density using a spectrophotometer set at 586 nm [66].

### Measurement of reduced glutathione (GSH) concentration

A cuvette containing the liver homogenate and GSSG was put into the spectrophotometer. It was combined with NADPH. 340 nm was the absorbance measurement. By subtracting the optical density of the blank tube from the sample net optical density, which was calculated using K•PO_4_ instead of GSSG [67].

### Assessment of catalase (CAT) activity

Methanol and H_2_O_2_ were added to the liver homogenate in the KH_2_PO_4_-NaOH buffer. The catalase enzyme was added, and the enzymatic process was initiated for 20 min at 20 °C. In order to halt the enzymatic action, KOH solution was then added. After adding Purpald, a further incubation was set up for ten minutes at 20 °C. Potassium periodate was added to KOH to oxidize the reaction product between formaldehyde and Purpald. At 550 nm, the absorbance was calculated using a spectrophotometer [68].

### Assessment of superoxide dismutase (SOD) activity

Tris–HCl was mixed with the liver homogenate, and pyrogallol was then added. By observing the increase in optical density at 420 nm, it was possible to quantify the change in optical density per minute. Using a blank tube, the inhibition % for the samples was calculated [69].

### Assessment of collagen type-1 and 4-hydroxyproline content in hepatic tissue

#### Collagen type-1 content

Biotin diluted in assay buffer (Tris, BSA, and Tween-20) was applied to a 96-well streptavidin plate and incubated for 30 min. 20 µL of sample or peptide calibrator + 100 µL of conjugated monoclonal antibody was incubated. Finally, 100 µL of tetramethylbenzidine was added, and the plate was incubated for 15 min and shaken. The plate was washed by using Tris and NaCl after each incubation step. The TMB reaction was stopped using HCl and measured at 450 nm [70].

#### 4-Hydroxyproline content

Samples of liver were hydrolyzed for 24 h at 100 °C in hydrochloric acid. After cooling, the hydrolysate was centrifuged for ten minutes and neutralized with NaOH. The solution of chloramine T was combined with the supernatant. At RT, it was then incubated for ten minutes. After ten minutes, Ehrlich's solution was added. The optical density at 560 nm was measured after the final combination was incubated for 10 min at room temperature [71].

### Gene expression of α-smooth muscle actin (ASMA)

Liver tissues were homogenized. Then, total RNA was extracted and measured with a dual spectrophotometer. The ASMA gene expression was quantitatively analyzed using qRT-PCR. For cDNA synthesis, cDNA reverse transcriptase was added to 1000 ng of extracted RNA. Afterward, cDNA was amplified as follows: enzyme activation for 10 min at 95 °C, then 40 cycles of 15 s at 95 °C, 20 s at 55 °C, and 30 s at 72 °C [72]. The data were normalized concerning the β-actin gene as an internal control gene.

Primer sequences for the ASMA gene:

**Table:** 

Gene symbol	Primer sequence
ASMA	5′-GAGCGTGGCTATTCCTTCGTG-3′ (F) 5′-CAGTGGCCATCTCATTTTCAAAGT-3′ (R)

### Histopathological examination of fibrotic tissue with special stain

Masson trichrome stain was performed for liver fibrosis scoring. After thioacetamide induction for 9 weeks, liver tissues were taken. The collected liver tissues were embedded, fixed, and divided into sections before being stained with hematoxylin and eosin (H&E) stain, and after that, slides were stained with Masson trichrome. Images were randomly taken from each region, and ImageJ software (version 1.6.0_20) was used to analyze the fibrotic area quantitatively [73].

### Statistical analysis

In triplicate (n = 3), the data were displayed as means and standard deviation (SD). Data were tested for normality using the Shapiro–Wilk test. Since the data met the assumptions of normality, a parametric test was used, one-way ANOVA with Tukey post-hoc testing.

GraphPad Prism version 8 was used to represent the data in graphs, and one-way ANOVA with Tukey post-hoc testing was used to assess the data's statistical significance. The means with SD of every parameter were compared pairwise using the Tukey post-hoc test, and the outcomes were displayed at the top of each bar, where p < 0.0001, a: Substantial difference when compared to the control group. b: Notable in comparison to the group that was treated with thioacetamide. c: Significant in contrast to the group that received thioacetamide with BM-MSCs. d: Significant in contrast to the group that received thioacetamide with AD-MSCs.

## Results

### Effect of treatments using BM-MSCs, AD-MSCs, and L-MSCs on liver function tests

**Fig. 1:**
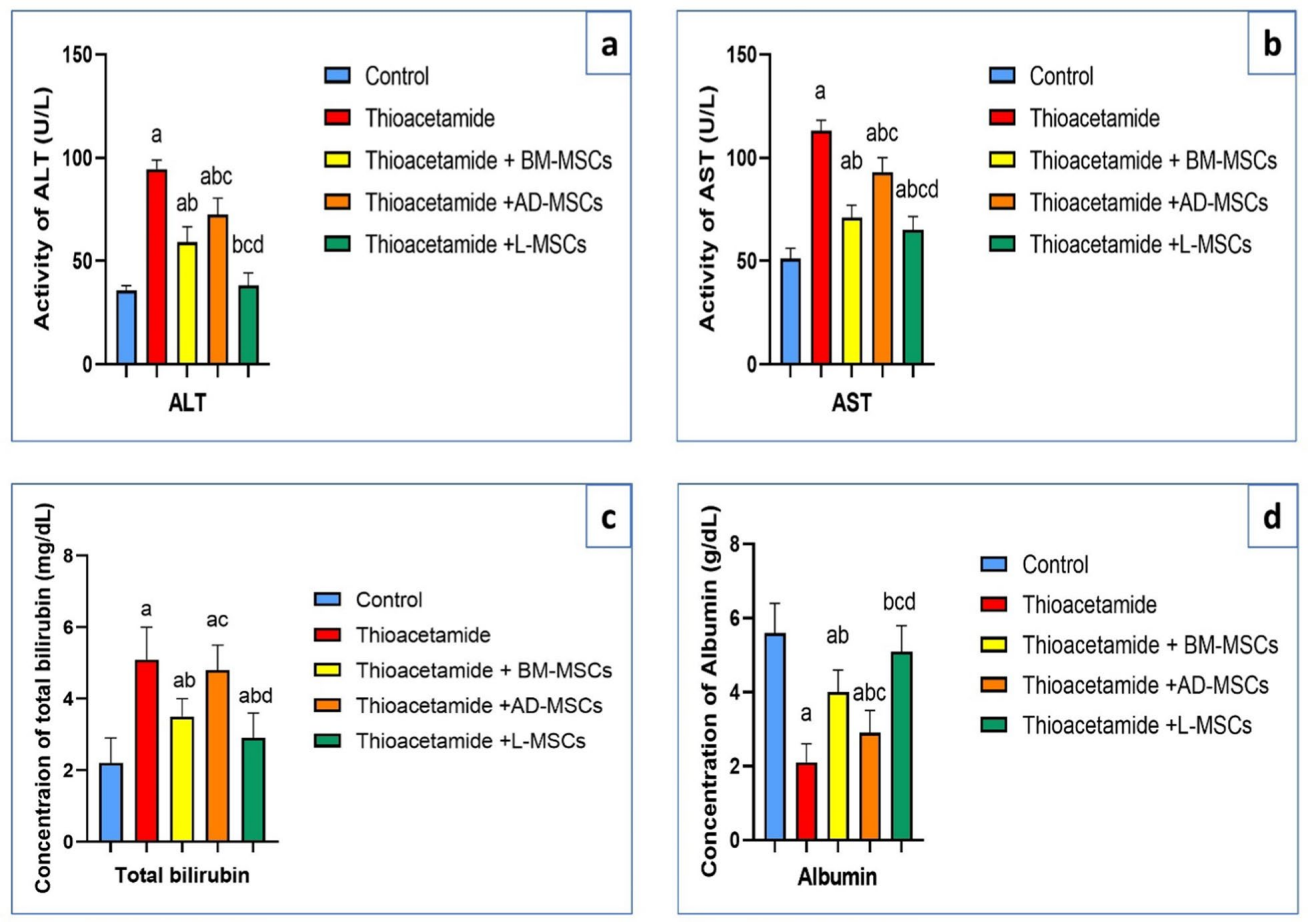
Effect of treatments using BM-MSCs, AD-MSCs, and L-MSCs on liver function tests: **a** The graph demonstrates the effect of various MSCs on the activity of the ALT enzyme. **b** The graph demonstrated the effect of various MSCs on the activity of the AST enzyme. **c** The graph demonstrated the effect of various MSCs on the concentration of total bilirubin. **d** The graph demonstrated the effect of various MSCs on the concentration of albumin

Table 1 showed that the thioacetamide group had the highest activity of ALT and AST enzymes; the least activity of them appeared in the thioacetamide + L-MSCs group, with a significant decrease compared to the thioacetamide + BM-MSCs and thioacetamide + AD-MSCs groups (Fig. 1a, b). As well, the highest concentration of total bilirubin appeared in the thioacetamide group, and the lowest concentration of total bilirubin appeared in the thioacetamide + L-MSCs group, with a significant decrease compared to the thioacetamide + BM-MSCs and thioacetamide + AD-MSCs groups (Fig. 1c). In contrast, the lowest concentration of albumin appeared in the thioacetamide group, and the most significant increase appeared in the thioacetamide + L-MSCs group (Fig. 1d).

**Table 1:** Liver function tests among the studied groups

Parameters	Control	Thioacetamide	Thioacetamide + BM-MSCs	Thioacetamide + AD-MSCs	Thioacetamide + L-MSCs
ALT (U/L)	35.7 ± 2.3	94.5 ± 4.4^a^	59 ± 7.6^ab^	72.4 ± 8^abc^	38.2 ± 6^bcd^
AST (U/L)	51 ± 5	113 ± 5.2^a^	71 ± 6^ab^	93 ± 7.1^abc^	65 ± 6.6^abcd^
Total bilirubin (mg/dL)	2.2 ± 0.7	5.1 ± 0.9^a^	3.5 ± 0.5^ab^	4.8 ± 0.7^ac^	2.9 ± 0.7^abd^
Albumin (g/dL)	5.6 ± 0.8	2.1 ± 0.5^a^	4.0 ± 0.6^ab^	2.9 ± 0.6^abc^	5.1 ± 0.7^bcd^

All data are represented as mean ± SD with P < 0.0001

^a^Significant difference compared to the control group

^b^Significant compared to the Thioacetamide group

^c^Significant compared to the (Thioacetamide + BM-MSCs) group

^d^Significant compared to the (Thioacetamide + AD-MSCs) group

### Effect of treatments using BM-MSCs, AD-MSCs, and L-MSCs on hepatic oxidative and anti-oxidative stress parameters

**Fig. 2:**
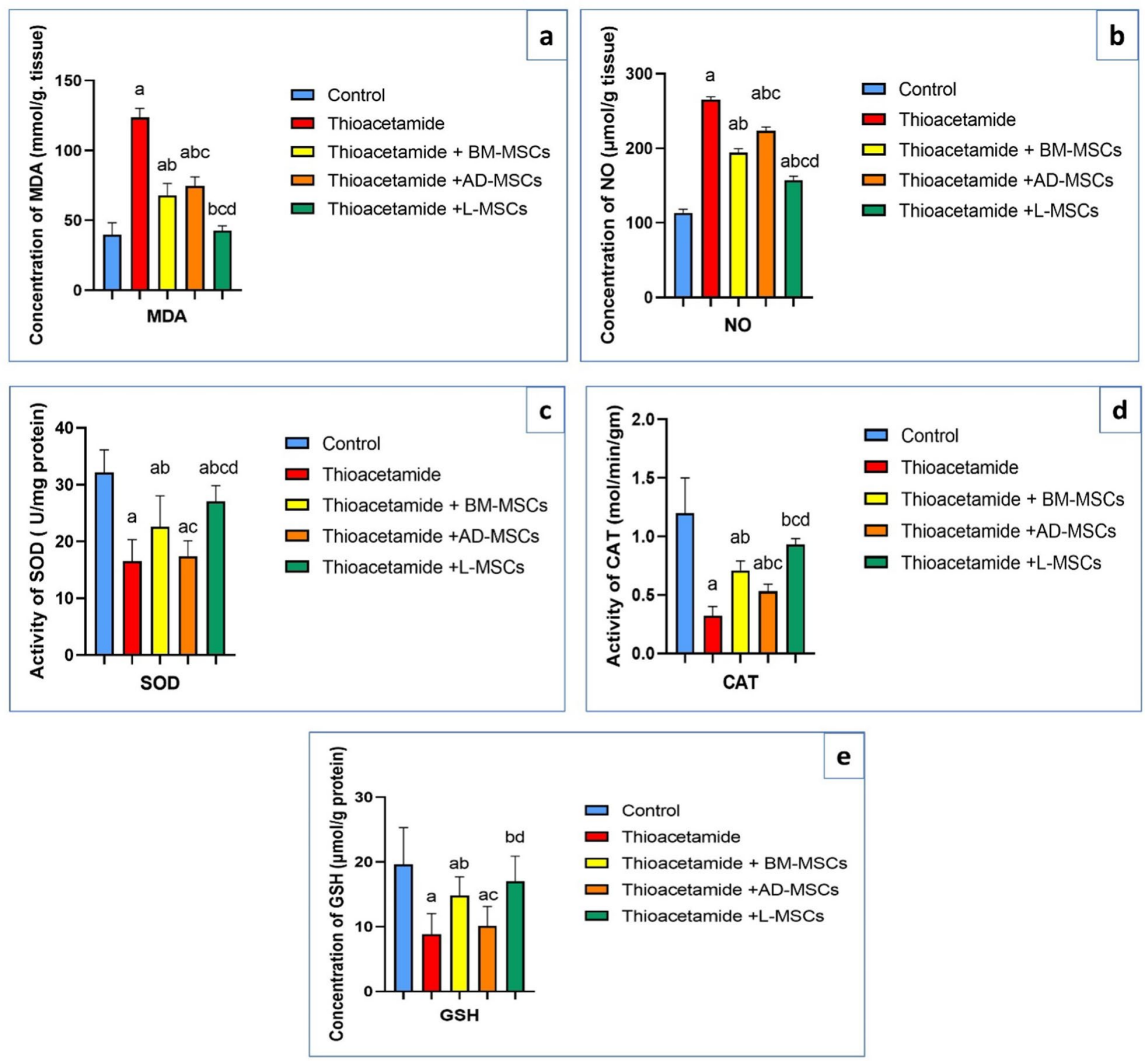
Effect of treatments using BM-MSCs, AD-MSCs, and L-MSCs on the antioxidant status and oxidative stress: **a** The graph illustrates the impact of different Mesenchymal Stem cell therapies on MDA levels. **b** The graph illustrates the impact of different Mesenchymal Stem cell therapies on NO levels. **c** The graph illustrates the impact of different Mesenchymal Stem cell therapies on SOD activity. **d** The graph illustrates the impact of different Mesenchymal Stem cell therapies on CAT activity. **e** The graph illustrates the impact of different Mesenchymal Stem cell therapies on GSH levels

Table 2 showed that the thioacetamide group had the greatest amount of MDA and NO. The level of MDA returned to normal in the thioacetamide group after treatment with L-MSCs, and that group's level of NO showed a significant decrease (Fig. 2a, b). The thioacetamide group had the lowest levels of SOD and CAT, but SOD levels went back to normal after treatment with L-MSCs, and CAT levels increased significantly. The least significant increase of SOD and CAT appeared in the thioacetamide + AD-MSCs group (Fig. 2c, d). The amount of GSH was significantly decreased in the thioacetamide group and returned to normal levels in mice with thioacetamide after treatment with L-MSCs, while it did not improve after treatment with AD-MSCs. As well, there is a significant increase in GSH level in the thioacetamide + BM-MSCs group (Fig. 2e).

**Table 2:** Hepatic oxidative and anti-oxidative stress parameters among the studied groups

Parameters	Control	Thioacetamide	Thioacetamide + BM-MSCs	Thioacetamide + AD-MSCs	Thioacetamide + L-MSCs
MDA (mmol/g tissue)	39.7 ± 8.6	123.6 ± 6.6^a^	67.9 ± 8.5^ab^	74.6 ± 6.4^abc^	42.7 ± 3.3^bcd^
NO (μmol/g)	113.4 ± 4.8	265.2 ± 3.8^a^	194.2 ± 5.4^ab^	223.8 ± 4.7^abc^	157.1 ± 5.2^abcd^
SOD (U/mg protein)	32.1 ± 4	16.5 ± 3.8^a^	22.6 ± 5.4^ab^	17.4 ± 2.7^ac^	27 ± 2.8^abcd^
CAT (mol/min/gm)	1.2 ± 0.3	0.32 ± 0.08^a^	0.71 ± 0.08^ab^	0.53 ± 0.06^abc^	0.93 ± 0.05^bcd^
GSH (μmol/g protein)	19.62 ± 5.7	8.8 ± 3.2^a^	14.8 ± 2.9^ab^	10.1 ± 3^ac^	17 ± 3.9^bd^

All data are represented as mean ± SD with P < 0.0001

^a^Significant difference compared to the control group

^b^Significant compared to the Thioacetamide group

^c^Significant compared to the (Thioacetamide + BM-MSCs) group

^d^Significant compared to the (Thioacetamide + AD-MSCs) group

### Effect of treatments using BM-MSCs, AD-MSCs, and L-MSCs on collagen type-1 and hydroxyproline content

**Fig. 3:**
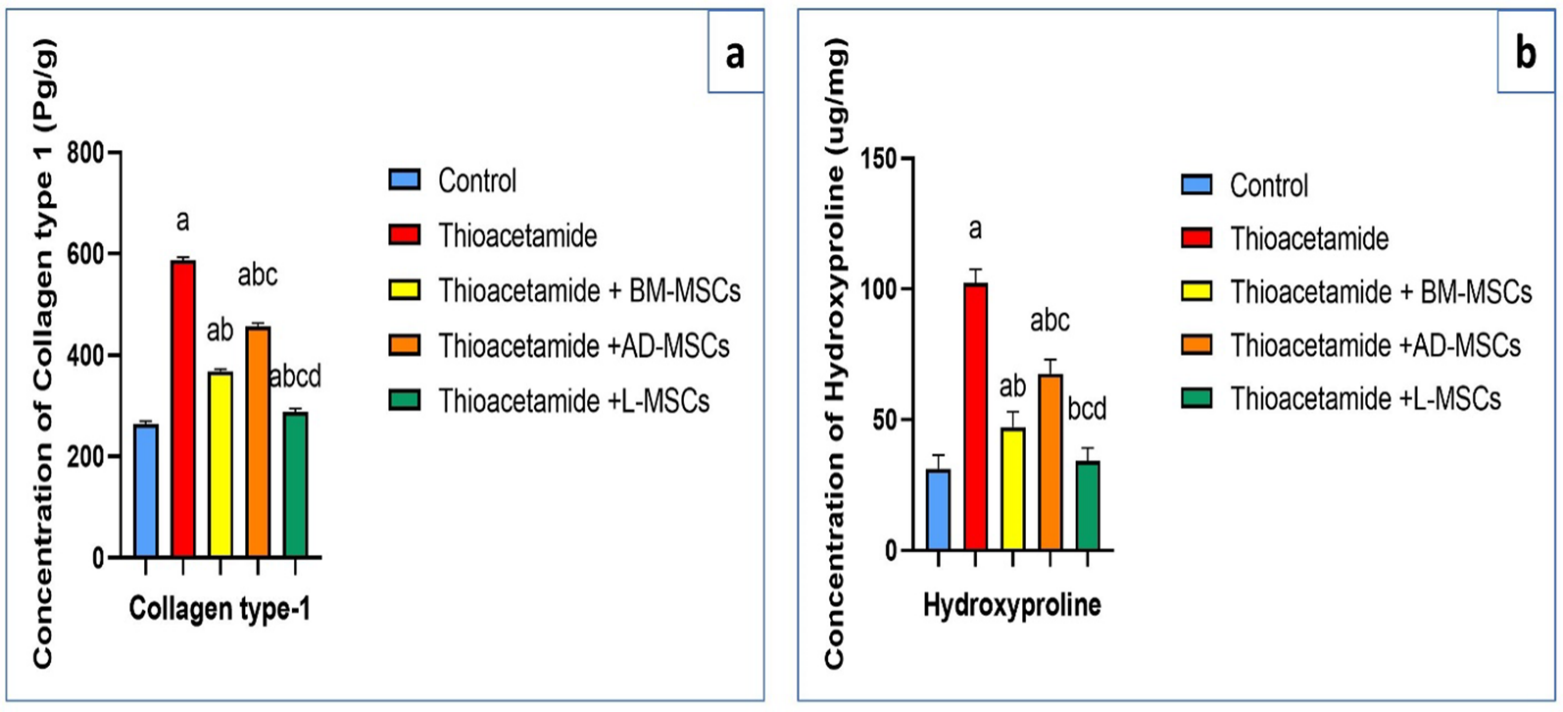
Effect of treatments using BM-MSCs, AD-MSCs, and L-MSCs on levels of the collagen type-1 and 4-hydroxyproline in different groups: **a** The graph illustrates the impact of different Mesenchymal Stem cell therapies on the content of collagen type-1. **b** The graph illustrates the impact of different Mesenchymal Stem cell therapies on the content of 4-hydroxyproline

Table 3 showed that the highest content of collagen type-1 and hydroxyproline appeared in the thioacetamide group. The collagen type-1 content significantly decreased in all groups treated with BM-MSCs, AD-MSCs, and L-MSCs after induction of hepatic fibrosis with thioacetamide, but the most significant decrease in collagen type-1 content appeared in the thioacetamide + L-MSCs group (Fig. 3a). The hydroxyproline content in hepatic tissue returned to normal levels in the group treated with L-MSCs. As well, it significantly decreased in the group treated with BM-MSCs and AD-MSCs (Fig. 3b).

**Table 3:** Collagen type 1, hydroxyproline content levels among the studied groups

Parameters	Control	Thioacetamide	Thioacetamide + BM-MSCs	Thioacetamide + AD-MSCs	Thioacetamide + L-MSCs
Collagen type 1 (Pg/g)	264.3 ± 5.2	587.23 ± 5.5^a^	368.2 ± 4.1^ab^	456.8 ± 5.9^abc^	287.6 ± 7^abcd^
Hydroxyproline (ug/mg)	31.2 ± 5.2	102.2 ± 5.4^a^	47.2 ± 5.8^ab^	67.5 ± 5.6^abc^	34.2 ± 5^bcd^

All data are represented as mean ± SD with P < 0.0001

^a^Significant difference compared to the control group

^b^Significant compared to the Thioacetamide group

^c^Significant compared to the (Thioacetamide + BM-MSCs) group

^d^Significant compared to the (Thioacetamide + AD-MSCs) group

### Effect of treatments using BM-MSCs, AD-MSCs, and L-MSCs on the level of ASMA gene expression

**Fig. 4:**
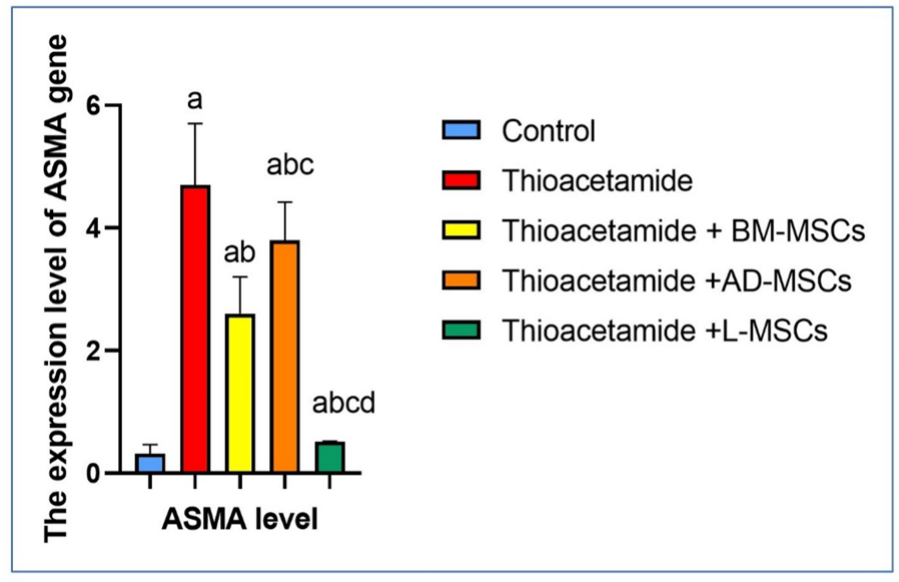
Effect of treatments using BM-MSCs, AD-MSCs, and L-MSCs on the ASMA expression: the graph illustrates the impact of different Mesenchymal Stem cell therapies on the expression of the ASMA gene

Table 4 and Fig. 4 illustrate that the thioacetamide group had the most significant increase in ASMA expression. However, the thioacetamide group that was treated with L-MSC showed the greatest decrease in ASMA expression. Additionally, ASMA expression was decreased with BM-MSC and AD-MSC therapy; however, the greatest improvement was shown with L-MSC treatment.

**Table 4:** The percentage of fibrotic area in histopathological examination among the studied groups

Parameters	Control	Thioacetamide	Thioacetamide + BM-MSCs	Thioacetamide + AD-MSCs	Thioacetamide + L-MSCs
Percentage of fibrotic area (%)	3.9 ± 0.62	43.2 ± 5.1^a^	22.3 ± 3.4^ab^	31.8 ± 3^abc^	7.2 ± 0.59^abcd^

All data are represented as mean ± SD with P < 0.0001

^a^Significant difference compared to the control group

^b^Significant compared to the Thioacetamide group

^c^Significant compared to the (Thioacetamide + BM-MSCs) group

^d^Significant compared to the (Thioacetamide + AD-MSCs) group

### Effect of treatments using BM-MSCs, AD-MSCs, and L-MSCs on the percentage of fibrotic area in histopathological examination by masson trichrome stain

**Fig. 5:**
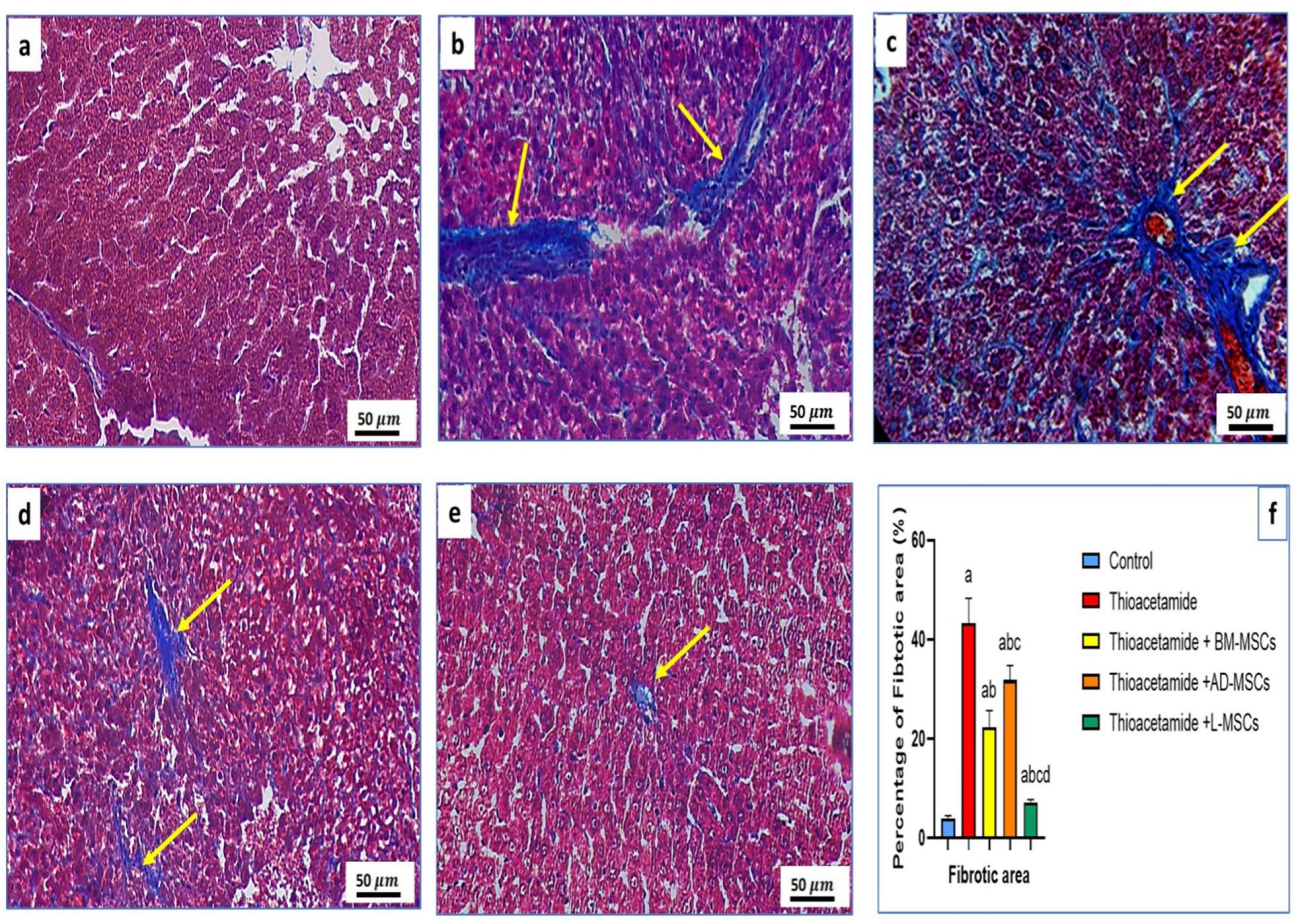
The histopathological examination of different groups treated with various MSCs: The histopathological examination of liver tissue from different groups stained with Masson trichrome to reveal the percentage of fibrotic area. **a** The control group, which was the normal group, had 3.9% of fibrotic area. **b** The positive control group, which received TAA only, had 43.24% of fibrotic area (yellow arrow). **c** The thioacetamide + BM-MSCs had 22.3% of fibrotic area (yellow arrow). **d** The thioacetamide + AD-MSCs had 31.8% of fibrotic area (yellow arrow). **e** The thioacetamide + L-MSCs had 7.2% of fibrotic area (yellow arrow). **f** The graph revealed the statistical analysis of the percentage of fibrotic area in different groups, and bars had different letters that mean there is a significant change

Table 5 and Fig. 5e showed that the group that received L-MSCs observed the greatest reduction in the percentage of fibrotic region, while Fig. 5b showed the greatest rise in the thioacetamide group. Furthermore, the percentage of fibrotic region was significantly lower in the BM-MSCs and AD-MSCs-treated groups than in the thioacetamide group (Fig. 5c, d).

**Table 5:** The expression of the ASMA gene among the studied groups

Parameters	Control	Thioacetamide	Thioacetamide + BM-MSCs	Thioacetamide + AD-MSCs	Thioacetamide + L-MSCs
ASMA level	0.32 ± 0.15	4.7 ± 1^a^	2.6 ± 0.6^ab^	3.8 ± 0.62^abc^	0.51 ± 0.01^abcd^

All data are represented as mean ± SD with P < 0.0001

^a^Significant difference compared to the control group

^b^Significant compared to the Thioacetamide group

^c^Significant compared to the (Thioacetamide + BM-MSCs) group

^d^Significant compared to the (Thioacetamide + AD-MSCs) group

## Discussion

In our study to induce liver fibrosis experimentally in rats, the rats were injected with TAA. To find out the effect of the different mesenchymal stem cells on ameliorating liver fibrosis, we assessed the liver function tests, including ALT and AST activities assessment, and the levels of total bilirubin and albumin. Our findings are in line with other studies [23, 74]. Numerous research studies have demonstrated that stem cells generate growth factors and cytokines to encourage regeneration, lower inflammation, and lessen the manufacture of extracellular matrix and the breakdown of excess ECM within the liver [75, 76]. Excessive collagen and other extracellular matrix protein buildup, together with a clinically significant reduction in liver function, are hallmarks of liver fibrosis [77]. Developing new hepatocytes to replace injured cells without generating too much extracellular matrix is the most efficient method of treating hepatic fibrosis [78]. Studies have shown that when there is severe liver injury, BM-MSCs can eventually create biliary cells and hepatic cells to help the healing process [79]. It has been studied whether BM-MSCs transplantation can help both acute and chronic liver diseases return to normal liver function [51]. According to earlier research, rats with damaged livers may have significantly decreased transaminase enzyme activity and total bilirubin levels and higher serum albumin levels after receiving BM-MSC transplantation [80, 81]. Our results are similar to those studies. The group that received L-MSCs showed the greatest improvement in the hepatic function test, which was consistent with earlier research [82], which reported that the MSCs have a great effect on the enhancement of liver function tests, decreasing ALT and AST activity, and increasing the albumin level.

As well, we assessed the antioxidant status in hepatic tissue homogenate among the different studied groups. The findings are in line with one theory, which is that BM-MSCs can scavenge reactive oxygen species (ROS). Which set off a series of events that led to hepatic fibrosis [83]. BM-MSCs emit a variety of chemicals, such as prostaglandin E2 [84]**,** which enhance antioxidant status, reduce oxidative stress, have anti-inflammatory properties, and reduce the necrosis of hepatic cells [85]. These crucial roles of BM-MSCs might be in charge of the post-transplant recovery of liver markers. In our study, the decreased levels of MDA and NO as well as the increased level of GSH and activities of SOD and CAT in BM-MSCs are aligned with previous studies [81, 86] that found there was a significant decrease in the MDA and NO levels in BM-MSC-treated groups compared to other groups due to the ability of those cells to release some chemicals that have antioxidant activity. In line with earlier research, the L-MSC-treated group had the greatest enhancement in antioxidant status and a reduction in oxidative stress [82], whose findings shown that L-MSCs can improve antioxidant status with a significant increase in SOD, CAT, and hepatic glutathione reductase enzyme activities, as well as a significant decrease in free radicals.

In this study, we evaluated collagen type-1 and 4-hydroxyproline content, as well as ASMA expression, to examine the anti-fibrotic characteristics of MSCs. The results prove the BM-MSCs' anti-fibrotic behavior and align with earlier studies [81, 87], which found that BM-MSCs could develop into functional hepatocytes and subsequently create several growth factors that helped to alleviate liver damage, and that the fibrosis region vanished following therapy and no collagen development was seen in the group treated with BM-MSCs. One sign of activated HSCs is the liver's expression of ASMA [88]. Additionally, excessive collagen and 4-hydroxyproline, which is the main composition of collagen type 2, and other ECM protein buildup due to activation of fibroblasts and hepatic stellate cells, as well as clinical liver function deterioration, are characteristics of liver fibrosis [77]. So, the content of collagen type-1 and 4-hydroxyproline highly significantly increases in the non-treated group, as well as the upregulation of ASAM expression. However, the most anti-fibrotic activities and the greatest decrease of the collagen type-1 and 4-hydroxyproline content, as well as the greatest downregulation of ASMA expression, appeared in the L-MSCs-treated group. This proves the ability of the L-MSCs to restore normal architecture of the liver and decrease the liver fibrosis, which corresponds to the earlier studies [89, 90], which reported that L-MSCs contain unique exosomes, vascular endothelial growth factor (VEGF), hepatocyte growth factor (HGF), antiapoptotic factor IL-6, and anti-inflammatory cytokines like IL-10. These substances help treat liver fibrosis by stopping HSC activation, which leads to HSC cell death and the breakdown of fibrous tissue. Additionally, L-MSCs generate matrix metalloproteinases that break down the extracellular matrix and have fibrinolytic action.

The golden tool for confirming the anti-fibrotic activity of different MSCs, we stained the histopathological section with Masson trichrome stain, which highlights the collagen fibers, and calculated the percentage of fibrotic area by ImageJ software. The outcome supported the BM-MSCs' anti-fibrotic properties and was consistent with other research [81, 91, 92], which reported that liver damage can be lessened by BM-MSCs producing a number of growth factors and cytokines after differentiating into functional hepatic cells. While the most significant increase in the percentage of fibrotic area appeared in the non-treated group and the most significant decrease in the percentage of fibrotic area appeared in the L-MSCs-treated group, which approves the anti-fibrotic activity of L-MSCs more than other MSCs, and that corresponds to a previous report [93], which reported that the anti-inflammatory properties of MSCs also lessen hepatocyte damage, which prevents hepatic stellate cells (HSCs) from activating, causing HSC death and fibrinolysis to occur. Additionally, another study claims that MSCs alter macrophage orientation to make them anti-inflammatory, boost the production of matrix metalloproteinases to decrease extracellular matrix, and improve macrophage capacity to phagocytose hepatocyte debris (during which macrophages increase prodegenerative factors) [94].

Even though this study gives valuable insights into the comparative effects of MSCs derived from different sources on thioacetamide-induced liver fibrosis, it has some limitations. The study lacked blinding throughout the treatment, which may introduce bias. Also, the study was limited to a single time point for the administration of MSCs. Upcoming studies should contain larger animal models, several time points, and extended period follow-up for better evaluating the therapeutic durability of MSCs. A comprehensive investigation of the underlying molecular mechanisms and verifying these results in large animal models or human clinical trials will additionally enhance the translational relevance of this study.

## Conclusions

Finally, we clarified in this study the role of different MSCs in ameliorating liver fibrosis, and cell therapy is an important way to promote tissue regeneration. Our study proved the greatest effect of the L-MSCs in the improvement of the liver functions, the antioxidant status, and the highly effective anti-fibrotic activity of those cells, which is even greater than BM-MSCs.

## Data Availability

Data supporting this study is included within the article.

## Abbreviations

AD-MSCs: Adipose mesenchymal stem cells
AKT: Protein kinase B
ALT: Alanine transaminase
ASCs: Adult stem cells
ASMA: α-Smooth Muscle Actin
ALB: Albumin
AST: Aspartate transaminase
BM-MSCs: Bone marrow mesenchymal stem cells
BSA: Bovine serum albumin
CAT: Catalase enzyme
cDNA: Copy deoxyribonucleic acid
CO_2_: Carbon dioxide
DMEM: Dulbecco's Modified Eagle Medium
ECM: Extracellular matrix
ESCs: Embryonic stem cells
FBS: Fetal bovine serum
FeCl_3_: Ferric chloride
GSH: Reduced glutathione
GSSG: Glutathione disulfide
H&E: Hematoxylin and eosin
H_2_O_2_: Hydrogen peroxide
HCl: Hydrochloric acid
HGF: Hepatocyte growth factor
HSCs: Hepatic stellate cells
IL-10: Interleukin 10
IL-6: Interleukin 6
iPSCs: Induced pluripotent stem cells
JAK/STAT: Janus kinase/signal transducers and activators of transcription
K•PO_4_: Potassium phosphate
KC: Kupffer cell
KH_2_PO_4_: Potassium dihydrogen phosphate
KOH: Potassium hydroxide
LDH: Lactate dehydrogenase
L-MSCs: Liver mesenchymal stem cells
LSD: Least significant difference
MDA: Malonaldehyde
MEM: Minimum essential media
MMPs: Metalloproteinases
MSC: Mesenchymal stem cell
NaCl: Sodium chloride
NAD: Nicotinamide adenine dinucleotide
NAFLD: Non-alcoholic fatty liver disease
NaOH: Sodium hydroxide
NASH: Non-alcoholic steatohepatitis
NO.: Nitric acid
PBS: Phosphate buffer saline
PCR: Polymerase chain reaction
PDGF: Platelet-derived growth factor
Pen/Strip: Penicillin/streptomycin
PI3K: Phosphatidylinositol 3-Kinase
qRT-PCR: Quantitative reverse transcription polymerase chain reaction
RAF: Rapidly accelerated fibrosarcoma
RAS: Rat sarcoma
RNA: Ribonucleic acid
ROS: Reactive oxygen species
RT: Room temperature
SMA: Smooth muscle actin
SOD: Superoxide dismutase enzyme
TAA: Thioacetamide
TIMPs: Tissue inhibitors of MMPs
TMB: 3,3′,5,5′-Tetramethylbenzidine
UM-SCs: Umbilical cord stem cells
VCl_3_: Vanadium (III) chloride
VEGF: Vascular endothelial growth factor
